# Blunted modulation of hierarchical brain organization in opioid use disorder

**DOI:** 10.21203/rs.3.rs-9889932/v1

**Published:** 2026-07-10

**Authors:** Dardo Tomasi, Peter Manza, Şükrü Barış Demiral, Natasha Giddens, Annabelle Belcher, Weizheng Yan, Kylee B. Miller, Faith N. Veenker, Christina Lildharrie, Michele-Vera Yonga, Sarah Abey, Leyla Brucar, Michaelene VanDine, Gene-Jack Wang, Nora D. Volkow

**Affiliations:** 1 National Institute on Alcohol Abuse and Alcoholism, National Institutes of Health, Bethesda, Maryland, USA.; 2 Kahlert Institute for Addiction Medicine, University of Maryland, Baltimore, Maryland, USA.; 3 Northwell Health, Division of Child and Adolescent Psychiatry, Zucker Hillside Hospital and Cohen’s Children’s Medical Center, Hofstra University, Glen Oaks, NY, USA.; 4 Department of Psychiatry, University of Maryland School of Medicine, Baltimore, MD, USA; 5 Department of Neurology, Stony Brook Medicine, Stony Brook, NY, USA

## Abstract

Opioid use disorder (OUD) is associated with persistent catecholaminergic dysfunction, but its impact on large-scale cortical organization remains unclear. We used pharmacological resting-state fMRI and functional gradient mapping to test whether OUD alters catecholaminergic modulation of cortical hierarchy. Fifty-three individuals with OUD and 40 healthy controls underwent resting-state fMRI after placebo and methylphenidate. Under placebo, principal and secondary cortical gradients showed canonical organization with no group differences. In controls, methylphenidate compressed the dynamic range of the principal gradient while preserving its spatial topology, indicating state-dependent modulation of the unimodal-transmodal hierarchy. This response was attenuated in OUD and further reduced among participants receiving methadone or buprenorphine. Methylphenidate improved visual attention in controls but not OUD, and gradient modulation predicted attentional benefit only in controls. Gradient features also predicted years of opioid use. These findings identify blunted catecholaminergic modulation of cortical hierarchy as a clinically relevant systems-level feature of OUD.

## INTRODUCTION

Opioid use disorder (OUD) is a chronic and relapsing disorder marked by compulsive drug seeking and dysregulation of reward circuitry^1–3^, and is associated with persistent impairments in cognitive control, including self-regulation and executive dysfunction, disrupted motivation, and alterations in sensorimotor processing^4,5^. Neuroimaging studies have demonstrated that OUD is associated with widespread alterations across cortical and subcortical systems, extending beyond classic mesolimbic reward circuitry to include frontoparietal, somatomotor, and association networks^6–8^. These distributed abnormalities suggest that OUD involves disruptions in large-scale brain organization rather than isolated regional deficits. However, most prior work has focused on region- or network-specific effects, leaving open the question of how OUD impacts the hierarchical architecture that organizes functional interactions across the entire brain.

Functional connectivity gradients provide a compact framework for characterizing the hierarchical functional organization of the human brain by capturing continuous axes of variation in large-scale functional connectivity patterns^9^. The principal gradient reflects a unimodal-to-transmodal hierarchy, spanning primary sensory and motor cortices to heteromodal association regions such as the default mode network (DMN), while secondary gradients capture orthogonal axes of functional specialization^9^. These gradients are highly conserved across individuals and datasets, yet remain sensitive to cognitive state, development, and neuromodulatory influences^10–12^. As such, gradient-based approaches oier a powerful means to quantify both trait-like and state-dependent alterations in macroscale brain functional organization.

Dopamine (DA) plays a central role in regulating large-scale cortical communication^13–16^. Pharmacological agents that alter dopaminergic tone have been shown to influence functional connectivity and network segregation^14,17–19^, suggesting that DA contributes to the dynamic tuning of hierarchical brain functional organization. Methylphenidate (MP), a drug that inhibits DA and norepinephrine reuptake, provides a well-characterized experimental probe of dopaminergic modulation and has been shown to induce state-dependent changes in resting-state functional connectivity^17,19–22^. However, it remains unclear how acute dopaminergic challenge impacts diierentially the scaling and topology of functional connectivity gradients in individuals with OUD, in whom dopaminergic signaling is chronically altered^23,24^.

Pharmacological treatments such as methadone and buprenorphine are central to the clinical management of OUD and are known to exert complex effectson dopaminergic function^25–27^. While these medications reduce craving, withdrawal symptoms, relapse risks and overdoses, evidence suggests that they may only partially normalize DA signaling and associated neural processes^25,26,28^. Neuroimaging studies have reported both persistent and treatment-related alterations in brain function among medicated individuals with OUD, highlighting substantial heterogeneity within this population^6,29^. How medications for OUD (MOUD) shape state-dependent modulation of large-scale cortical organization under acute dopaminergic challenge remains poorly understood, underscoring the need to explicitly consider medication status when examining macroscale brain dynamics in OUD.

Previous work from our group demonstrated that MP selectively compressed the principal functional gradient in healthy adults, with effects localized to the somatomotor (SMM) network and was associated with striatal D1 and D2 receptor availability^22^. This identifies SMM cortex as a primary site of dopaminergic modulation of hierarchical brain organization providing an a priori framework for testing how such modulation is altered in OUD. In the present study, we used resting-state functional MRI gradient mapping to investigate large-scale cortical organization in OUD after placebo and after MP. We hypothesized that the baseline macroscale cortical gradients in individuals with OUD would be disrupted when compared to controls and that the compression of the principal gradient with MP would also be attenuated along with a blunting of MP’s attention enhancing effects.

## RESULTS

### Sample characteristics and data quality

Within the OUD group (n=53), 18 participants were unmedicated, 11 were receiving buprenorphine, and 24 were receiving methadone at the time of scanning (Table 1). Participants completed two resting-state fMRI sessions after placebo (PL) and after MP on separate days in a randomized, counterbalanced order, with MP administered as a single 60-mg oral dose.

Due to scheduling or tolerability issues, 15 OUD participants did not complete the MP session, and 2 OUD participants did not complete the PL session; in the control group (n=40), 2 participants did not complete the MP session. Mean framewise displacement (FD) was analyzed using a linear mixed-effects (LME) model with group and drug condition as fixed effects and participant as a random intercept. Intelligence quotient (IQ) scores were lower in OUD participants (P<0.001), while sex, age, and race did not differ between groups (Table 1). Controls exhibited lower FD than OUD participants (P<0.001), and a significant group-by-drug interaction indicated that the reduction in FD under MP was greater in OUD than in controls (P=0.005; Table 1). FD and IQ were included as covariates in all subsequent analyses to account for these effects. MP was well tolerated, with no serious adverse events reported; mild and transient effects consistent with its known pharmacological profile were observed and did not differ systematically across groups or treatment subtypes. These results indicate that subsequent analyses were not confounded by demographic differences, motion, session order, or data quality.

### Baseline differences in macroscale cortical gradients

To assess trait-like alterations in large-scale cortical organization, we compared principal and secondary functional connectivity gradients between OUD participants and healthy controls under placebo conditions using the LME model with sex, age, body mass index (BMI), IQ, and FD covariates. Both gradients exhibited the canonical unimodal-to-transmodal organization in each group, with highly similar spatial topographies (Supplementary Fig. S1). The LME analysis did not reveal a significant main effect of group on either the principal or secondary gradient at placebo baseline after false-discovery rate (FDR) correction for multiple comparisons. These findings indicate that, at rest under placebo, macroscale cortical hierarchy is largely preserved in OUD relative to controls.

### Methylphenidate eCects on gradients: within-group modulation

**Figure 1.**
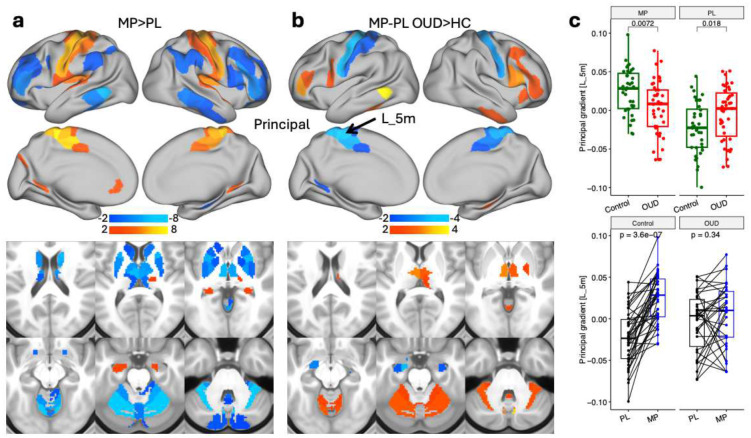
Effects of methylphenidate on the principal functional gradient. (**a**) Main effect of drug (MP>PL) across participants. (**b**) Group-by-drug interaction highlighting regions where MP–PL effects differed between individuals with opioid use disorder (OUD; n=53) and healthy controls (HC; n=40); the arrow indicates the area in the somatomotor cortex used for summary analyses (L_5m). Cortical and subcortical effects are shown on surface renderings and axial slices, respectively; warm and cool colors denote relative increases and decreases in gradient values (t-score map). (**c**) Top: Boxplots of residual principal gradient values at L_5m under MP and PL conditions for control and OUD groups (median, interquartile range, and individual data points shown) after removal of confounds (age, sex, race, IQ, BMI, and framewise displacement), with P values for between-group comparisons. Bottom: Paired plots showing within-subject MP–PL changes for each group, with lines connecting individual participants and P values indicating within-group effects.

LME analyses revealed a significant main effect of drug and a group-by-drug interaction for both the principal and secondary gradients (Fig. 1). MP reduced the dynamic range of the principal gradient overall, covering all 28 parcels of the *a priori* MP-sensitive network (MPsN; Fig. S2) as well as in an extended MPsN (P_FDR_<0.05, corrected for multiple comparisons using the false discovery rate; 0.31<Cohen’s d<0.85; Supplementary Tables S1 and S2 and Fig. 1a), but this compression was attenuated in OUD relative to controls, most prominently within bilateral SMM areas and anterior cingulum (P_BH-FDR_<0.05, using the Benjamini–Hochberg FDR correction for multiple comparisons in the a priori MPsN; 0.54<Cohen’s d<0.92; Table S1). Individuals with OUD also exhibited weaker compression of the principal gradient in parietal (supramarginal gyrus), inferior temporal, dorsolateral prefrontal cortices, and amygdala, and stronger compression in the thalamus and anterior cerebellum (P<0.05, uncorrected; Fig. 1b and Tables S1–2). In contrast, MP exerted minimal effects on the secondary gradient, and no significant group differences were observed for MP-related changes in this gradient (Fig. S3).

**Figure 2.**
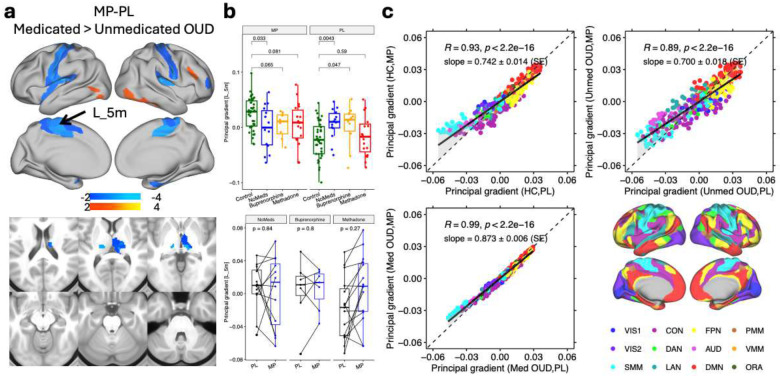
Medication-related di<erences in MP e<ects on the principal functional gradient in OUD. (**a**) Regions showing greater MP–PL eWects on the principal gradient in medicated compared with unmedicated individuals with OUD; the arrow indicates the somatomotor region used for summary analyses (L_5m). Cortical surfaces and axial slices display significant eWects, with warm and cool colors denoting relative increases and decreases (t-scores). (**b**) Top: Boxplots of residual principal gradient values at L_5m under MP and PL after removal of confounds (age, sex, race, BMI, IQ, and FD) for controls (n=40), unmedicated OUD participants (n=18), and OUD participants receiving buprenorphine (n=11) or methadone (n=24), with individual data points and P values for between-group comparisons. Bottom: Paired plots showing within-subject MP–PL changes for each OUD subgroup, with connecting lines and P values indicating within-group eWects. (**c**) Scatterplots depict parcel-wise correspondence between placebo (PL) and methylphenidate (MP) sessions for the principal functional gradient in healthy controls (top left), medicated individuals with OUD (bottom left), and unmedicated individuals with OUD (top right). Solid lines indicate least-squares fits and dashed lines denote the identity line. Insets illustrate network topography on cortical surfaces. Each point represents a parcel (n=438), colored by canonical functional network aWiliation^30^: Visual (VIS1, VIS2), Somatomotor (SMM), Cingulo-Opercular (CON), Dorsal Attention (DAN), Language (LAN), Frontoparietal (FPN), Auditory (AUD), Default-Mode (DMN), Posterior Multimodal (PMM), Ventral Multimodal (VMM), and Orbito-AWective (ORA).

We next examined whether MOUD contributed to heterogeneity in gradient responses within the OUD group. LME analyses contrasting medicated (methadone or buprenorphine) and unmedicated OUD participants revealed significant effects of MOUD on macroscale cortical gradients (Fig. 2). Relative to unmedicated participants, individuals receiving MOUD exhibited lower compression of the principal gradient in somatomotor, opercular, insular, and cingulate cortex areas (P_BH-FDR_<0.05; 0.35<Cohen’s d<1.28; Table S1), mirroring the differences between OUD and HC, and no significant effects on the secondary gradient. No significant differences in MP-related compression of the principal gradient were observed between healthy controls and unmedicated OUD participants in any brain region.

Across groups, principal gradient values under MP remained strongly correlated with PL values at the parcel level, indicating preservation of overall gradient topology despite drug-related modulation (Fig. 2c). High correlations were observed in controls, medicated and unmedicated OUD participants (R>0.89; P<2.2E-16), with systematic deviations from the identity line consistent with MP-related compression of gradient values. In contrast, the secondary gradient showed near-complete correspondence between MP and PL sessions across all groups (R>0.96; Fig. S4), indicating minimal drug-related perturbation of this orthogonal axis of functional organization.

### Visual attention

**Figure 3.**
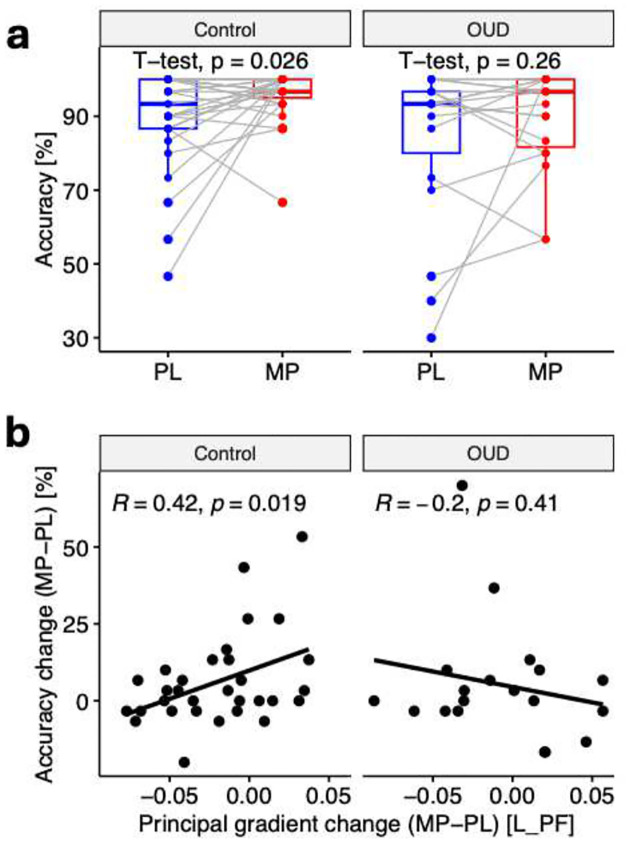
Effect of MP on visual attention performance. (**a**) Visual attention accuracy under placebo (PL) and methylphenidate (MP) in 31 controls and 19 individuals with OUD. Lines indicate within-subject pairs; paired t-test results are shown.

We tested whether MP-related improvements in visual attention accuracy was associated with MP-related compression of the principal gradient in the supramarginal gyrus (PF; inferior parietal cortex, Brodmann area 40)^22^. Among the 31 controls who completed the ball-tracking task, visual attention (VA) accuracy (averaged across 2- and 3-ball runs) was significantly higher following MP compared with PL (paired t-test, P=0.026; Fig. 3a). In contrast, VA accuracy did not differ between MP and PL in the 19 OUD participants (P=0.26). Accuracy was significantly lower in the MOUD group (P<0.02, t-test; MP and PL sessions included), whereas no significant differences were observed between the unmedicated OUD group and healthy controls (Fig. S5). Consistent with this pattern, MP-related improvements in VA accuracy were positively associated with MP-induced changes in principal gradient strength in prefrontal regions among controls (P<0.015; ANOVA controlling for age, sex, and race), but not among OUD participants (P = 0.40; Fig. 3b).

### Prediction of years of opioid use

We used PLS regression with leave-one-out cross-validation to assess the associations between functional gradients and age or years of opiate use. In each fold, a PLS model was trained on N−1 participants from one condition and tested on the held-out participant in the other condition. This was done in both directions, PL→MP and MP→PL, to assess cross-condition generalization^22^.

**Figure 4.**
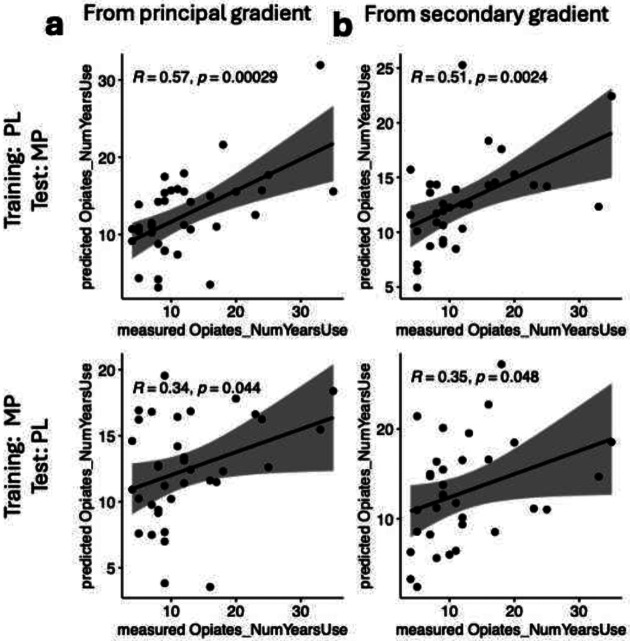
Prediction of opioid-use duration. (**a–b**) PLS prediction of years of opioid use from functional gradients across 36 OUD participants, controlling for age. Measured versus predicted values are shown for models based on the principal (**a**) and secondary (**b**) gradients across pharmacological conditions. Shaded areas denote 95% confidence intervals.

The principal gradient significantly predicted age in both controls and individuals with OUD (P < 0.04), whereas the secondary gradient was not associated with age in either group. In contrast both the principal and the secondary gradients predicted the number of years of opiate use in the OUD participants (Fig. 4). The association between years of use and the principal gradient remained significant after regressing out the linear effect of age (R>0.44, P<0.01).

## DISCUSSION

Here, we assessed large-scale cortical brain functional organization in OUD after placebo and after MP which we used as a dopaminergic challenge. Contrary to our hypothesis that baseline macroscale cortical gradients would diier in OUD, the principal and secondary gradients under placebo did not diier between groups, suggesting preserved large-scale topology at rest. On the other hand, dopaminergic challenge with MP, which selectively compressed the principal gradient while largely preserving its spatial organization, was attenuated in individuals with OUD particularly within MP-sensitive somatomotor regions. The attenuated compression was moderated by MOUD status, highlighting heterogeneity in dopaminergic responsiveness within OUD.

The principal functional gradient captures a dominant axis of cortical organization spanning unimodal somatomotor systems and distributed transmodal association networks, reflecting progressive integration across large-scale functional systems^9,31^. Reductions in the dynamic range of this gradient suggest a shift toward more homogeneous or locally constrained functional interactions, consistent with theoretical and empirical models linking neuromodulatory influences to changes in network integration and segregation^32,33^. DA has been proposed to tune the integration–segregation balance of cortical networks by modulating synaptic gain and signal-to-noise ratios, thereby influencing the extent of distributed communication across the cortex^32–35^. In this framework, MP-induced increases in catecholaminergic tone may favor more locally synchronized processing at the expense of long-range diierentiation, resulting in the observed compression of the unimodal–transmodal hierarchy^22^. Importantly, the preservation of overall gradient topology and the stability of the secondary gradient indicate that dopaminergic modulation primarily alters the scaling of intrinsic functional organization rather than its fundamental spatial layout^22^, highlighting a dissociation between state-dependent neuromodulatory effects and trait-like aspects of cortical hierarchy.

The attenuated MP effects observed in individuals with OUD suggest that chronic opioid exposure alters the brain’s dynamic capacity for dopaminergic modulation of large-scale functional organization. Long-term opioid use is associated with persistent adaptations in dopaminergic signaling, including reduced DA release and altered receptor availability^2,36–38^, which may constrain the dynamic range over which acute dopaminergic challenges can influence cortical hierarchy. Within the OUD group, MOUD status further shaped gradient responses, with medicated participants exhibiting reduced gradient compression relative to unmedicated individuals. This pattern is consistent with evidence that methadone and buprenorphine partially stabilize dopaminergic tone without fully restoring normative signaling dynamics^28 ,39^. Rather than reflecting normalization, these findings point to medication-dependent modulation of state responsiveness, underscoring substantial heterogeneity in dopaminergic function among individuals with OUD and highlighting the importance of considering treatment status when interpreting neuromodulatory effects on brain organization.

In controls, MP-related improvements in accuracy were associated with changes in principal gradient strength within parietal regions implicated in attentional control, consistent with prior work showing that catecholaminergic stimulation enhances frontoparietal network integration and supports goal-directed attention^40,41^. The absence of both behavioral enhancement and gradient–performance coupling in OUD participants may reflect altered cortical hierarchy–dopamine interactions or reduced dopaminergic responsivity, in line with PET studies demonstrating diminished striatal D2R availability and blunted dopamine signaling in opioid dependence^24^. Because prefrontal and frontostriatal control systems support adaptive decision-making and regulation of drug-cue-driven behavior^42,43^, impaired catecholaminergic modulation of cortical hierarchy may contribute to reduced cognitive flexibility and relapse vulnerability in OUD. Together, these findings suggest that intact gradient reconfiguration within parietal cortex may represent a neural mechanism through which MP enhances cognitive performance, one that appears compromised in OUD, potentially due to chronic dopaminergic dysregulation.

Consistent with prior lifespan studies, we found that the principal functional gradient, but not the secondary gradient, predicted age in both controls and individuals with OUD. Age-related compression or dedifferentiation along the principal unimodal-to-transmodal axis has been repeatedly observed in large population samples and multiscale aging analyses^44,45^. This pattern is also consistent with well-documented age-related changes in long-range functional connectivity within DMN and SMM regions^46^, and decreased system segregation^47,48^ in aging, further supporting the interpretation that normative hierarchical reorganization is preserved in OUD.

Although the overall sample size was substantial for a multimodal imaging study, subgroup analyses, particularly within medication categories, were necessarily limited and should be interpreted cautiously. MP increases both DA and norepinephrine availability, and thus the observed effects cannot be attributed exclusively to dopaminergic mechanisms. In addition, functional gradient measures are sensitive to head motion and preprocessing choices; although we rigorously controlled for motion and related confounds, residual effects cannot be entirely excluded. Finally, the findings may not generalize to other dopaminergic agents, doses, or cognitive states beyond resting conditions.

Together, these findings highlight altered dopaminergic regulation of hierarchical brain organization as a systems-level feature of OUD. We show that acute dopaminergic challenge can selectively modulate the scaling of the principal functional gradient, and that this modulation is attenuated in OUD. Thus, macroscale functional gradients can serve as sensitive markers of dopaminergic system integrity, and individual differences in gradient responsiveness may index variability in neuromodulatory capacity that is not captured by static measures of brain structure or baseline connectivity. Clinically, this framework may help characterize heterogeneity in treatment response, and to identify targets for interventions aimed at restoring adaptive brain dynamics. Future longitudinal and interventional studies combining molecular and systems-level imaging will be critical for determining whether normalization of dopaminergic signaling can re-establish flexible hierarchical organization and support recovery in OUD.

## METHODS

### Participants

Forty healthy adults and 53 individuals with OUD (for demographics see Table 1) provided written informed consent to participate in this study, which was approved by the Institutional Review Board (IRB) of the National Institutes of Health (NIH). To ensure precise assessment of reward-related neurobiological mechanisms, exclusion criteria included any history of substance abuse or dependence other than nicotine, major psychiatric disorders, neurological disease, or medical conditions known to affect cerebral or dopaminergic function (including cardiovascular, endocrine, oncological, or autoimmune disorders). To control for acute substance effects, participants underwent urine toxicology screening for cocaine, methamphetamine, amphetamines, opioids, cannabinoids, benzodiazepines, and barbiturates at study entry and on each study day. Healthy controls were required to test negative for all substances except nicotine, whereas individuals with OUD could test positive for opioids and other non-severe substances, provided they did not meet criteria for a current severe substance use disorder other than OUD. Additional exclusions comprised current use of prescription or over-the-counter medications and a history of head trauma associated with loss of consciousness exceeding 30 minutes. Portions of the imaging datasets from these cohorts have been reported previously using distinct analytical approaches^18,22,49–56^. However, the analyses and findings presented in this manuscript are entirely original.

Participants completed two imaging sessions conducted one week apart, each performed under either a 60-mg oral dose of MP or PL in a randomized, counterbalanced, double-blind design (Figs. 1a and 1b). MP was selected to robustly modulate dopaminergic signaling within mesocorticolimbic reward circuits. Resting-state fMRI data (21 minutes total across three consecutive scans) were obtained to capture MP- and PL-dependent alterations in intrinsic functional connectivity within reward-related brain networks.

### Visual attention

Thirty-one healthy controls and nineteen individuals with OUD completed a visually based, nonverbal visual attention (VA) task during fMRI scanning^57^. Participants were instructed to covertly track two or three designated target balls among ten moving balls. At the start of each 12-second trial, target balls were highlighted for 0.5 seconds, followed by one second of Brownian motion during which all balls moved randomly across the screen. Participants maintained central fixation while tracking the targets. After motion ceased, a new set of balls was briefly highlighted, and participants indicated via right index finger button press whether the highlighted balls matched the original targets. A 1-second response window was provided. The correct targets were then re-highlighted for 0.5 seconds to reorient attention before the next trial. Each participant completed two runs, one tracking two balls and one tracking three balls, with 15 trials per run (3 minutes each). Stimuli were presented on a BOLDscreen 32 liquid-crystal display (Cambridge Research Systems, UK).

### MRI acquisition

Participants completed two MRI sessions that included both structural and resting-state functional imaging on a 3.0 Tesla Magnetom Prisma scanner (Siemens Medical Solutions USA, Inc., Malvern, PA) equipped with a 32-channel head coil. Resting-state fMRI data were acquired using a multi-echo, multiband echo-planar imaging (EPI) sequence^58^ with an acceleration factor of three and anterior-to-posterior phase encoding. Acquisition parameters included a repetition time of 891 ms, echo times of 16, 33, and 48 ms, a flip angle of 57°, and coverage of 45 axial slices with a spatial resolution of 2.9 × 2.9 × 3.0 mm. To ensure reliable estimation of intrinsic functional connectivity, 471 consecutive volumes were collected over a single 7-minute resting-state run^59^ during which participants remained awake with eyes open. To minimize the influence of scanner-related acoustic noise on brain activity all participants wore ear protection. Visual fixation was maintained using a centrally presented cross displayed on a black background via an LCD projection system (BOLDscreen 32, Cambridge Research Systems, UK) in a dimly illuminated scanning environment. High-resolution anatomical data were acquired using a three-dimensional magnetization-prepared rapid gradient echo (MP-RAGE) sequence (TR = 2400 ms, TE = 2.24 ms, flip angle = 8 degrees) long with a variable flip angle turbo spin-echo sequence (Siemens SPACE; TR = 3200 ms, TE = 564 ms). Structural images were collected with isotropic 0.8-mm voxels, a field of view of 240 × 256 mm, a matrix size of 300 × 320, and 208 sagittal slices, providing detailed anatomical coverage for subsequent functional data alignment and analysis.

### MRI processing

Resting-state functional MRI data were preprocessed using fMRIPrep (version 24.0.1). Anatomical preprocessing included skull stripping, bias field correction, segmentation, and nonlinear spatial normalization to the MNI152NLin2009cAsym template. Functional images underwent motion correction, slice timing correction, and realignment to the mean functional image. Spatial normalization was performed using nonlinear warping. The TE-dependent analysis (tedana), an open-source Python tool designed for processing multi-echo fMRI data, was utilized to produce an adaptive T2* map and compute an optimally weighted combination of single-echo time series. Both volume-based and surface-based preprocessing were performed. Functional time series were resampled to the subject’s cortical surface using the FreeSurfer-derived white and pial surfaces and registered to the fsLR and fsaverage space for group-level analysis. For subsequent analysis, data were processed in 32k CIFTI format with 32,492 cortical vertices per hemisphere for a total of 91,282 grayordinates, which allows for high-resolution representation of both cortical and subcortical structures. Physiological and motion-related noise sources were identified and removed with nuisance regressors including global signals, white matter and cerebrospinal fluid signals and their temporal derivatives, temporal component-based physiological noise correction (tCompCor), six motion parameters (3 translations and 3 rotations), their temporal derivatives, and high-order polynomial trends. Framewise displacement (FD) and the root mean square (RMS) of voxelwise intensity differences between successive time points (e.g., DVAR) were computed, and treated as nuisance regressors, and volumes exceeding 0.5 mm FD and 0.5% DVARS were flagged to facilitate posterior denoising based on scrubbing. The final preprocessed images were resampled to 2 mm isotropic resolution in MNI space. Quality control metrics, including head motion, signal-to-noise ratio, and registration accuracy, were visually inspected to ensure preprocessing integrity.

### DiCusion embedding

We mapped principal functional connectivity gradients using a diiusion embedding approach^9^ implemented in Python (https://github.com/sensein/mapalign). Because applying diiusion embedding to dense, grayordinate-level connectomes is computationally intensive, we optimized the analysis by using parcellated connectomes, defined as Pearson correlation matrices between regions of interest, rather than computing full pairwise grayordinate connectivity. This low-dimensional representation substantially reduces computational complexity while preserving essential features of large-scale network organization, in line with prior research. Functional connectomes were constructed using the Integrated Cortico-Subcortical-Cerebellar Atlas (iCSC-438)^22^, which combines the 360 partitions of the multi-modal parcellation of the human cerebral cortex, the 50 thalamic and basal ganglia partitions of the Melbourne Subcortex Atlas, and the 28 cerebellar partitions of a probabilistic atlas of the human cerebellum. This atlas comprises 438 regions organized into 12 canonical networks 12 canonical network partitions^30^ in CIFTI space. For each scan, a 438 × 438 correlation matrix was computed using Connectome Workbench (https://www.humanconnectome.org/software/get-connectome-workbench). Adjacency matrices were generated by retaining the top 10% strongest positive connections per row, from which cosine similarity matrices were derived. Diiusion embedding was then applied to extract the first 10 low-dimensional connectivity gradients for each scan.

Because dimensionality reduction methods can yield gradients that vary in sign and component order across individuals, we aligned individual gradients to standardized group-level templates^22^. Group templates for the principal and secondary gradients were generated from the average parcellated connectome using the same embedding procedure. For each individual scan, the gradients showing the highest spatial correlation with these templates were identified as the principal and secondary gradients and used for subsequent group-level analyses^22^.

### A priori MP-sensitive ROI mask.

An a priori set of MP-sensitive parcels (MPsN) was defined using data from an independent within-subject simultaneous PET–MRI study of healthy adults (n=20) previously reported^55,56^. In that study, participants completed imaging sessions under placebo and following a single oral dose of MP (60 mg) in a randomized, counterbalanced, double-blind design. Functional connectivity gradients were computed using the same diiusion embedding methodology and the 438-ROI parcellation, and 28 parcels exhibiting significant MP-related compression of the principal gradient (MP vs placebo; FDR-corrected) were identified^22^. This independently defined regions-of-interest was used as an a priori mask for hypothesis-driven analyses in the present study (Fig. S4). No participants overlapped between that study and the current study.

### Statistical analysis

To assess group diierences and the eiect of MP on the strength of the connectivity gradients compared to PL, we implemented an LME modeling approach in Matlab. Separate LME models were fit for each gradient, with fixed effects for drug condition. Age, sex, race, BMI, IQ, and FD were included as covariates to control for potential confounding effects. A random intercept for each participant was included to account for within-subject dependencies across the three resting-state scans collected in each drug condition. Model selection and inference were based on likelihood ratio tests and restricted maximum likelihood estimation. Hypothesis-driven analyses were restricted to an a priori set of MP-sensitive ROIs (Fig. S2). This ROI set corresponded to regions exhibiting significant MP-related compression of the principal gradient in healthy adults^22^. In the current cohort, group-by-drug interaction effects were tested within this predefined ROI subset, with multiple-comparison correction applied across 28 parcels using Benjamini–Hochberg false discovery rate (BH-FDR). Whole-parcellation analyses across all 438 ROIs were additionally performed as secondary analyses.

### PLS analysis

Partial Least Squares (PLS) regression was conducted in R using the pls package to examine whether low-dimensional gradient components predicted years of opiate use. Both predictor (gradient components) and outcome (age and years of opiate use) variables were standardized (z-scored) prior to analysis to ensure comparability across measures. The optimal number of PLS components was selected via cross-validation by identifying the model that minimized prediction error. Model performance was assessed using the coefficient of determination (R^2^) and root mean squared error (RMSE).

## Supplementary Material

Supplementary Files

This is a list of supplementary files associated with this preprint. Click to download.

- SupplementaryMaterial.docx

## Data Availability

The data that support the findings of this study are available from the corresponding author upon reasonable request, subject to institutional approvals, data-use agreements, and applicable participant privacy protections.

## Code availability

Custom code used to perform the functional gradient analyses is available from the corresponding author upon reasonable request. The analyses also relied on publicly available software packages, as described in the Methods.
